# Ovarian Cancer Metastasis to the Larynx: A Case Report and Review of the Literature

**DOI:** 10.1155/2020/1543129

**Published:** 2020-08-03

**Authors:** Chad Purcell, Ayham Al Afif, Martin Bullock, Martin Corsten

**Affiliations:** ^1^Division of Otolaryngology-Head and Neck Surgery, Dalhousie University, QEII Health Sciences Centre, 3rd Floor Dickson Building, 5820 University Avenue, Halifax, Nova Scotia, Canada B3H 1Y9; ^2^Department of Pathology, Dalhousie University, Sir Charles Tupper Medical Building, Room 11B, 5850 College Street, P.O. Box 15000, Halifax, Nova Scotia, Canada B3H 4R2

## Abstract

Laryngeal secondary malignancies are rare, and most spread locoregionally from hypopharyngeal or thyroid primaries. Metastasis of ovarian carcinoma to the larynx is extremely rare. A 65-year-old woman with a history of high grade serous ovarian carcinoma was undergoing carboplatin chemotherapy for recurrence. She presented with progressive dysphagia and hoarseness; a computer tomography (CT) scan demonstrated bilateral necrotic lymphadenopathy and hypopharyngeal fullness. A hypopharyngeal mass was confirmed on examination, and operative biopsy identified it as high-grade serous ovarian. To our knowledge, this report describes the second immunohistochemically proven metastatic ovarian cancer detected in the larynx in the world literature.

## 1. Introduction

Direct extension of primary head and neck malignancies to the larynx is well described, including squamous cell carcinoma (SCC) of hypopharynx and papillary carcinomas of the thyroid gland and hemopoietic malignancies [1]. However, metastatic spread to the larynx from distant primary sites is very rare, accounting for only 0.09 to 0.40% of all laryngeal tumors [2]. We were only able to identify three cases of a secondary laryngeal neoplasm identified in a patient with ovarian cancer in the literature [3–5]. This article presents a case of immunohistochemically proven ovarian cancer metastases to the larynx.

## 2. Case Report

A 65-year-old woman was referred to our care for urgent evaluation of a several month history of progressive dysphagia to solids, globus sensation, and hoarseness. Her past medical history was significant for hypertension, hypothyroidism, gastroesophageal reflux disease, rheumatoid arthritis, and pancreatitis. She was diagnosed with stage IVB high-grade serous carcinoma (HGSC) of the ovary 4 years prior to her presentation. She underwent a hysterectomy and bilateral salpingo-oophorectomy with adjuvant chemotherapy with carboplatin, Taxol, and bevacizumab. Her disease unfortunately recurred 2 years later, with metastases to the bowel and liver. She was subsequently restarted on carboplatin therapy. Other medications include levothyroxine, hydrochlorothiazide, rivaroxaban, pantoprazole, and dexamethasone. She is an ex-smoker with a 15-pack-year history.

**Figure 1:**
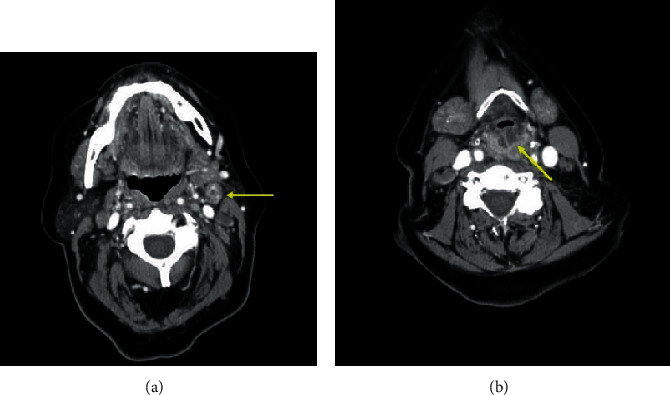
Axial nonenhanced computed tomography scan of the neck. (a) Necrotic cervical lymph nodes at level IIa and (b) submucosal edema of the supraglottis.

**Figure 2:**
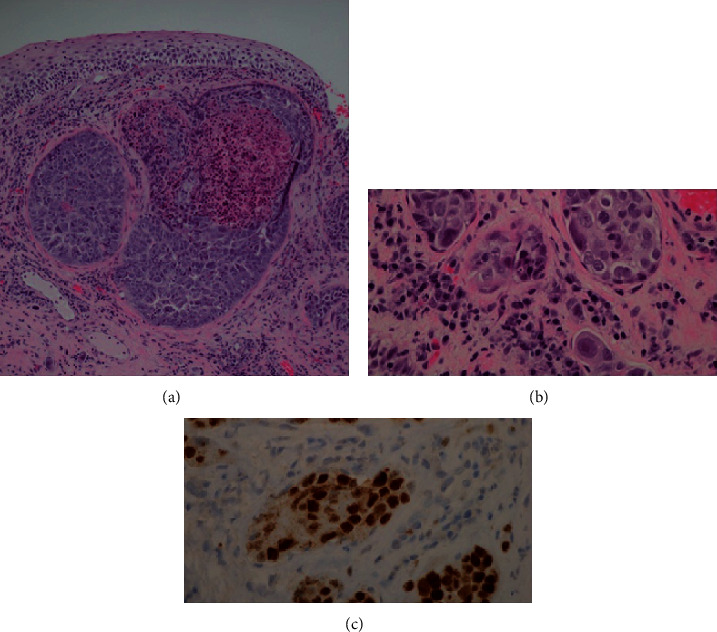
Microscopic features of arytenoid tissue samples. (a) Arytenoid mucosa showing large nests of malignant cells in the lamina propria with normal overlying surface epithelium (H&E 100x). (b) High power image showing small nests of cells plugging lymphatics in the lamina propria. Note the variability in nuclear size, irregularly shaped nuclei, and prominent nucleoli typical of ovarian serious carcinoma (H&E 400x). (c) Immunohistochemical stain for Pax8, showing nuclear positivity in the tumor cells, consistent with metastatic serous carcinoma (Pax8 400x).

On examination, she was hoarse with no stridor. Palpable cervical lymphadenopathy was appreciable in level II bilaterally and level IV on the right. Flexible nasopharyngoscopy demonstrated marked epiglottic edema. There were appreciable hypomobility and fullness in the right vocal fold. The right aryepiglottic fold demonstrated submucosal irregularities, and the left aryepiglottic fold and arytenoid were edematous. Fine needle aspiration (FNA) of a left level II mass was performed at the time. An urgent CT scan was arranged (Figure 1). Despite high-dose steroid therapy, the laryngeal edema rapidly progressed and she was consented for direct laryngoscopy and biopsy of the supraglottis (Figure 2).

Fine needle aspiration (FNA) of left neck level IIa node demonstrated malignant epithelial cells consistent with metastatic ovarian serous carcinoma, positive for CK7, PAX-8, p16, and p53 on immunohistochemical staining. CK20, estrogen receptor (ER), TTF-1, and CDX-2 were negative, with patchy equivocal staining for WT-1. Tissue biopsies of the right and left arytenoid areas demonstrated clusters of malignant glandular cells predominantly within lymphatics. These cells had morphological features very similar to the patient's prior ovarian carcinoma specimen from 4 years previously and were consistent with metastatic high grade ovarian serous carcinoma. An immunohistochemical was also performed on the biopsy specimens, with similar staining results to the FNA, but with more convincing WT1 positivity. The immunohistochemical results in both specimens overlapped those of the primary tumor. Given her overall poor prognosis with recurrent/metastatic ovarian cancer, the patient declined any further treatment of this laryngeal mass but provided consent to publish her case.

## 3. Discussion

Metastatic HGSOC was confirmed on pathological examination using known differentiating features of HGSOC [6]. A recent study found no obvious differences in IHC staining primary HGSC tumors and omental metastases [7]. The tumor in the larynx was largely intralymphatic, consistent with metastatic spread, and it had glandular features. There was no squamous differentiation to suggest a primary laryngeal squamous cell carcinoma. Markers of lung or thyroid origin (TTF1) and colonic origin (CDX2, CK20) were negative. In addition to the close morphologic similarity to the ovarian primary, the immunohistochemical demonstration of CK7, WT1, p53, and PAX8 are all consistent with metastatic ovarian serous carcinoma [8, 9]. Renal cell carcinoma—another tumor that may metastasize to head and neck sites [1, 10]—has some overlapping IHC findings with HGSOC but was excluded on the basis of morphology (renal cell carcinoma is typically a clear cell tumor) and the patient's history.

HGSOC is characterized by a spectrum of molecular diversity [6]. Primary tumors show more genomic similarities with distant metastases than contralateral ovarian tumors, and significant heterogeneity has been described among patients [11]. Precursor lesions for HGSOC tumors include STIC, SCOUT, and TP53 signatures [12]. *γ*-Synuclein (SNCG) has been implicated in tumor progression and metastasis in HGSOC [13]. Cheon et al. identified a 10-gene signature (AEBP1, COL11A1, COL5A1, COL6A2, LOX, POSTN, SNAI2, THBS2, TIMP3, and VCAN) that is associated with poor overall survival and metastasis in patients with HGSOC [14]. These 10 genes are responsible for collagen remodelling, suggesting that collagen remodelling may play a role in the pathophysiology of these tumors. The use of molecular testing to differentiate primary from metastatic HGSOC is an emerging field, and there is insufficient evidence to support the diagnostic utility of molecular testing to confirm the tumor source in such rare metastatic presentations of HGSOC.

Risk factors for laryngeal metastases are not well established, but males tend to have a greater predilection [1, 10]. Secondary malignancies present with symptoms mimicking primary malignancies of the hypopharynx or larynx, including hoarseness, stridor, dyspnea, dysphagia, otalgia, and globus sensation [15–17]. Symptomatic patients may present early, but presentation is often delayed compared to primary laryngeal malignancies, especially if the lesion is submucosal [18]. Laryngeal metastases are indicative of widespread metastatic disease.

Secondary neoplasms of the larynx can present with mucosal lesions or with invasion into a cartilaginous laryngeal framework. Laryngeal secondary malignancies most commonly involve the supraglottis, followed by the subglottis and glottis [1, 10, 19, 20]. Metastatic lesions involving multiple subsites at the same site have been described as transglottic [10].

The exact mechanism of metastatic spread to the larynx from distant primaries remains elusive. Distant metastasis may occur via hematogenous and/or lymphatic spread from distant primary tumors [21]. Hematogenous spread may occur via the “vena cava” route or retrograde spread from the valve-less vertebral venous plexus, also known as Batson's plexus [22, 23]. Batson's plexus features frequent reversals of flow which may provide a pathway for metastatic spread that does not involve the heart or lungs. The supraglottis has a very rich lymphatic network, while subglottic lymphatics are comparatively smaller and less compact [24]. This may explain why more supraglottic metastases have been reported. Lymphatic spread occurs via the deep cervical lymph nodes and can follow a typical drainage pattern or retrograde via anastomoses with superior laryngeal vessels that reach the supraglottic lymphatics through the thyrohyoid space [21, 25].

Laryngeal metastases are most commonly reported with melanoma, colorectal, renal, and prostate carcinomas [1, 10]. Less commonly, breast, lung, stomach, and ovarian cancer have been associated with laryngeal metastases [10]. Unlike other cancers, hematogenous spread is uncommon with HGSOC [26]. High-grade serous ovarian cancer (HGSOC) typically spreads by direct extension to the adjacent organs within the peritoneal cavity and extraperitoneal spread is rare. However, studies suggest that lymphatic spread to paraaortic and pelvic nodes may contribute to distant metastases [27].

Only three cases of ovarian cancer metastasizing to the larynx were discovered in a review of the literature [3–5]. Two reports identified subglottic lesions postmortem [3, 4]. Only one case described an immunohistochemically proven ovarian origin [4], but all three were biopsy proven [3–5]. One case presented a presumed metastatic lesion of the larynx in a patient with mesonephroid adenocarcinoma of the ovary, though diagnostic information was not provided [5]. Cullen reported on a case of a 71-year-old woman who presented with stridor and upper airway compromise, requiring urgent tracheostomy. She ultimately succumbed to heart and kidney failure, and an autopsy revealed ovarian and laryngeal tumors determined to be of the same anaplastic tumor subtype [3]. Freeland et al. reported an incidentally identified papillary serous cystadenocarcinoma found on autopsy with an unconfirmed subglottic lesion [4].

## 4. Conclusion

Metastatic lesions to the larynx most commonly manifest in the supraglottic or subglottic subsites [1, 10, 19, 20]. This is likely due to richer lymphatic and blood supply to these areas, compared to the glottis [10]. Laryngeal secondary malignancies are uncommon due to the terminal nature of blood and lymphatic circulation to the larynx [25]. The present case describes a metastatic supraglottic lesion immunohistochemically proven to be HGSOC. The literature on secondary neoplasms of the larynx is very sparse, and this study contributes to the body of evidence on this disease entity. This case highlights the importance of including secondary primary malignancies in the differential diagnosis of laryngeal masses.

## Additional Points

*Authorship*. CP, AA, and MC saw this patient in otolaryngology clinic, and operative biopsy was performed by MC. Pathological analysis and description of specimen and results was done by MB. The manuscript was drafted by CP and revised by AA and MC.
